# Supplementary figures and images for: Developmental hourglass: Verification by numerical evolution and elucidation by dynamical-systems theory
Source: PLoS Comput Biol. 2024 Feb 29;20(2):e1011867. doi: 10.1371/journal.pcbi.1011867 (PMC10903806; doi:10.1371/journal.pcbi.1011867)

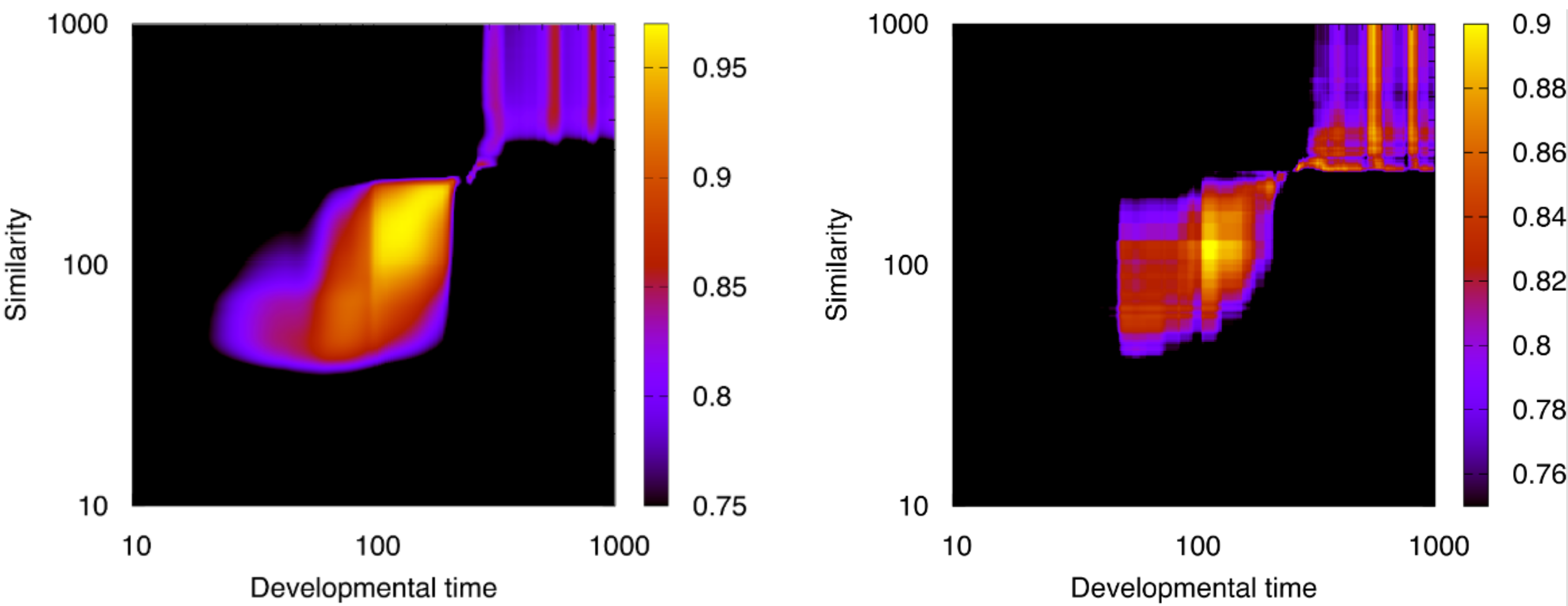

Supplement: S1 Fig — The similarity between the developmental dynamics of the two individuals shown in Fig 1A is represented as a heat map. The axes are the same as in Fig 1B, and the similarity is measured by Pearson’s coefficient on the left and Spearman’s rank coefficient on the right. (TIF) [file pcbi.1011867.s001.tif]

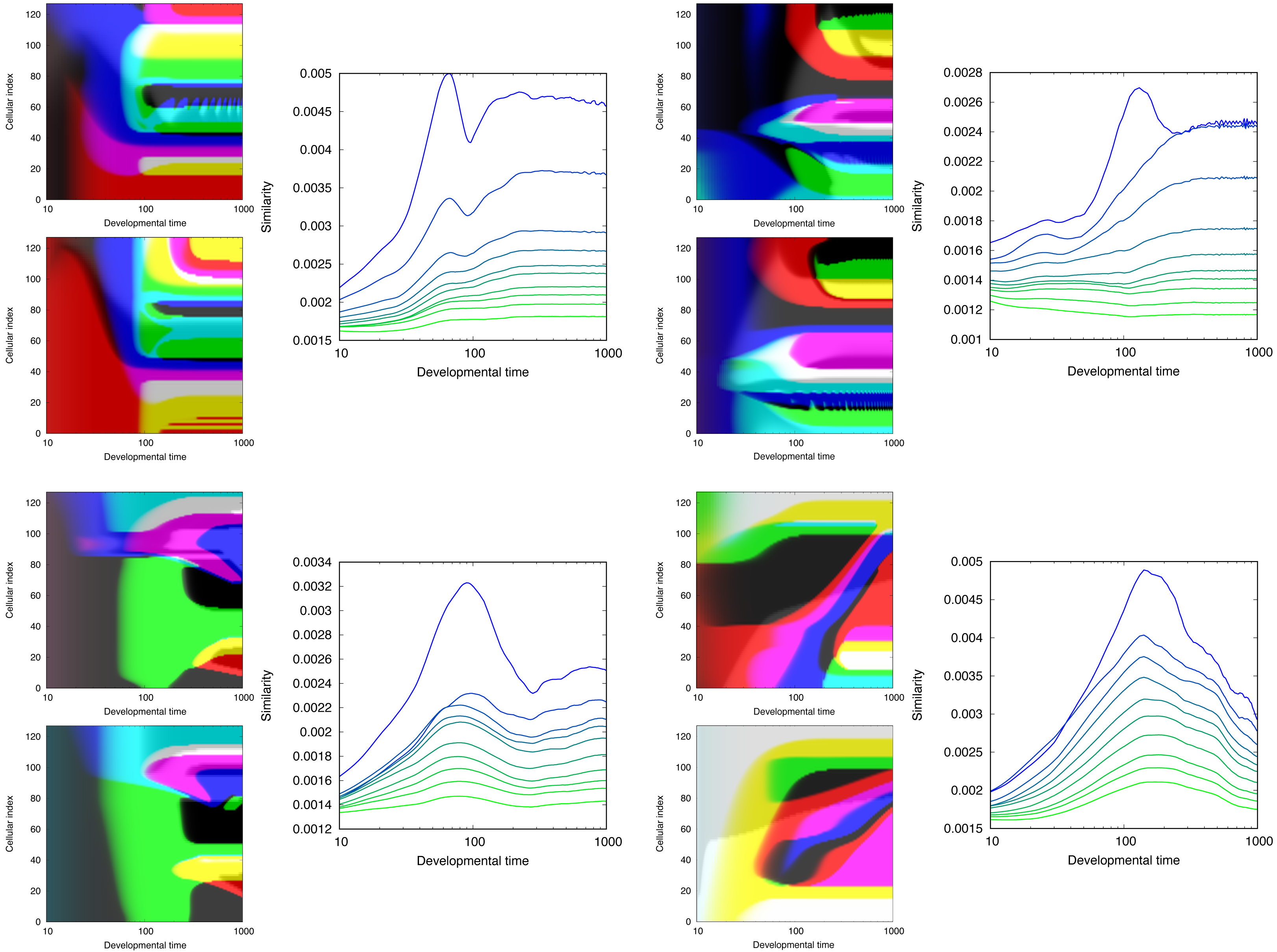

Supplement: S2 Fig — Four additional examples that show developmental hourglass are represented with developmental similarity. (Left) Two examples of gene expression pattern dynamics of evolved GRNs. The horizontal axis represents the developmental time, and the vertical axis represents the cellular index, i.e., space. (Right) The dependence of similarity is plotted against the developmental time, for species with different phylogenetic distance. The horizontal axis represents the developmental time, and the vertical axis represents the average of the similarity between the spatial patterns of gene expressions, in the same way as in Fig 2. The similarity is computed as in Fig 1C and averaged over pairs of individuals. The line colour denotes the phylogenetic distance of compared species departed from 900, 800,…, 100, 0th generations. The similarity calculated among the species that diverged every 100 generations, from the 900th to the 0th generation is plotted. (TIF) [file pcbi.1011867.s002.tif]

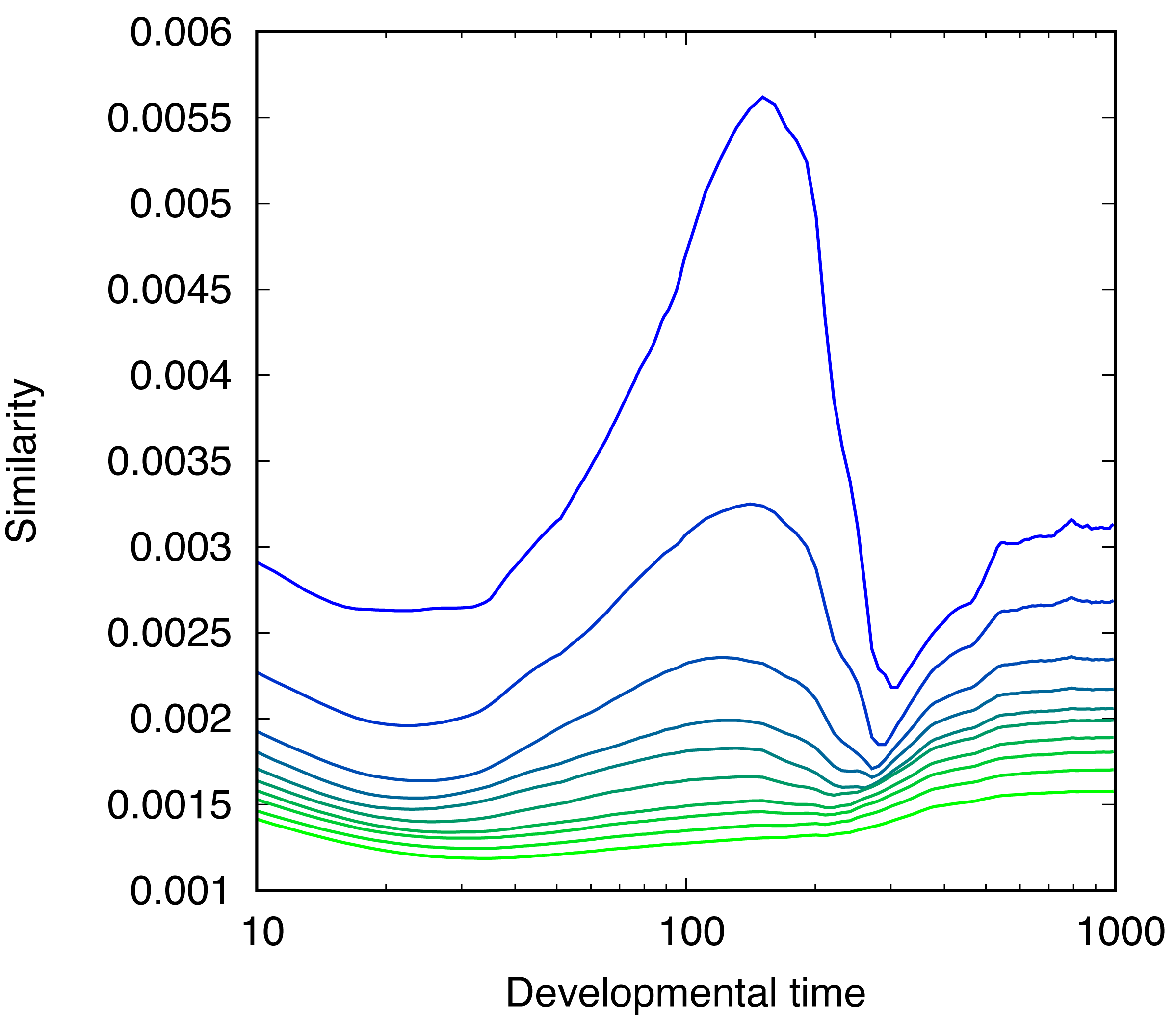

Supplement: S3 Fig — Similarities among the species that diverged from a common ancestor are plotted, using the data from the same simulation run as in Fig 2, but plotted with phylogenic distance per 100 generations. Axes are the same as in Fig 2(left). Each curve represents the similarity among species that have different length of shared generations in the evolutionary path. The top blue curve represents the similarity of species that share 900 generations out of total 1000 generations, and with each 100 generations decrease of the length of shared generations, results are plotted as different colours, from blue to green (see also Figs 1 and 2 for schematic representation). (TIF) [file pcbi.1011867.s003.tif]

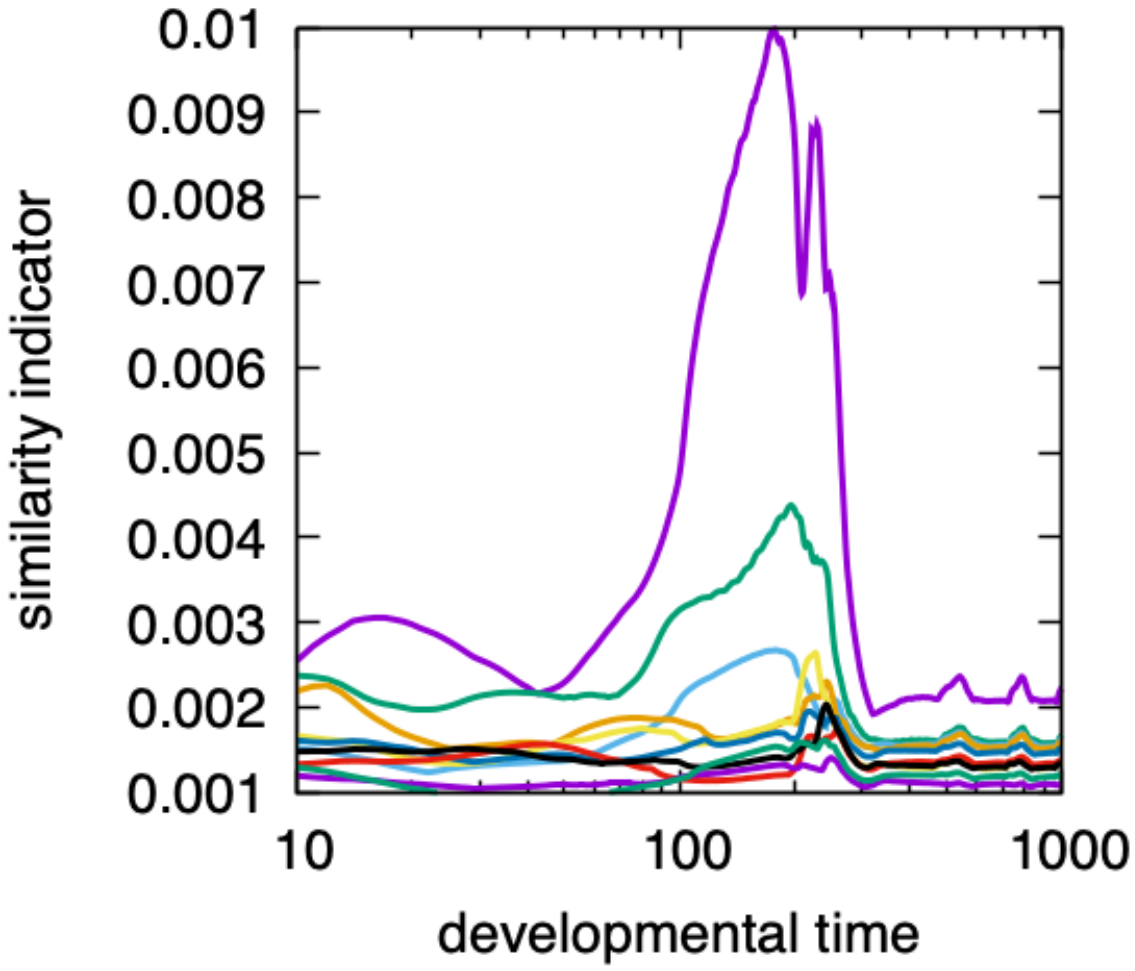

Supplement: S4 Fig — Similarities are plotted for the individual of Fig 2A1 with each 100-generation distant ancestor from the 900th to 0th generation. The horizontal axis is the developmental time of the evolved individual, and the vertical axis is the similarity indicator. The similarity is plotted in the same manner as in Fig 2C. The intermediate similarity peak is conserved from the 700th generation ancestor (i.e., 300 generations distant ancestor), which roughly corresponds with the slow gene control acquisition generation. (TIF) [file pcbi.1011867.s004.tif]

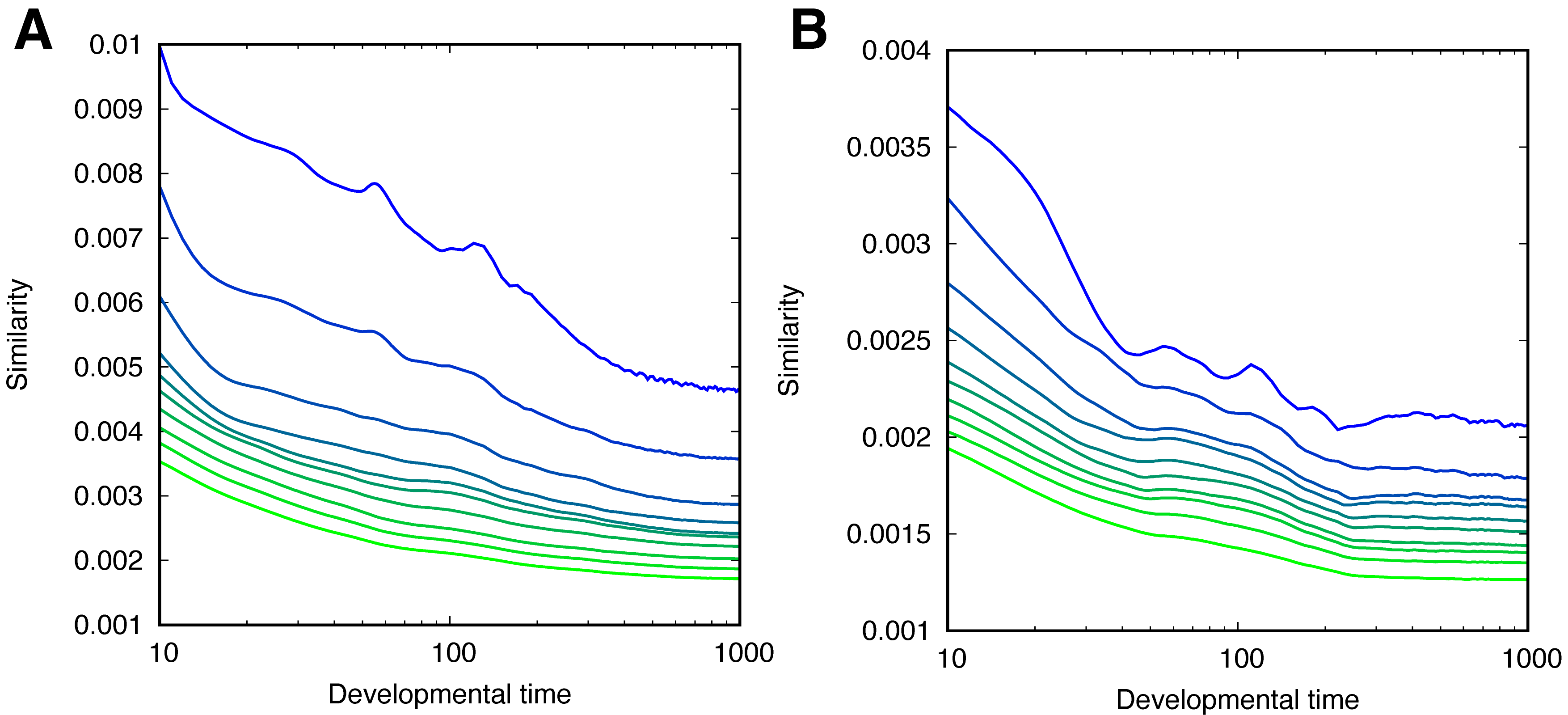

Supplement: S5 Fig — The relationship between phylogenetic distance and the value of the similarity indicator is represented as the same as Fig 2, SF2, and SF3 but with different noise levels on the initial condition. (A) An example of the case with the noise level on the initial condition σ = 0.01, which is 0.1 in the other figures. (B) An example of the case with the noise level on the initial condition σ = 0.001. (TIF) [file pcbi.1011867.s005.tif]

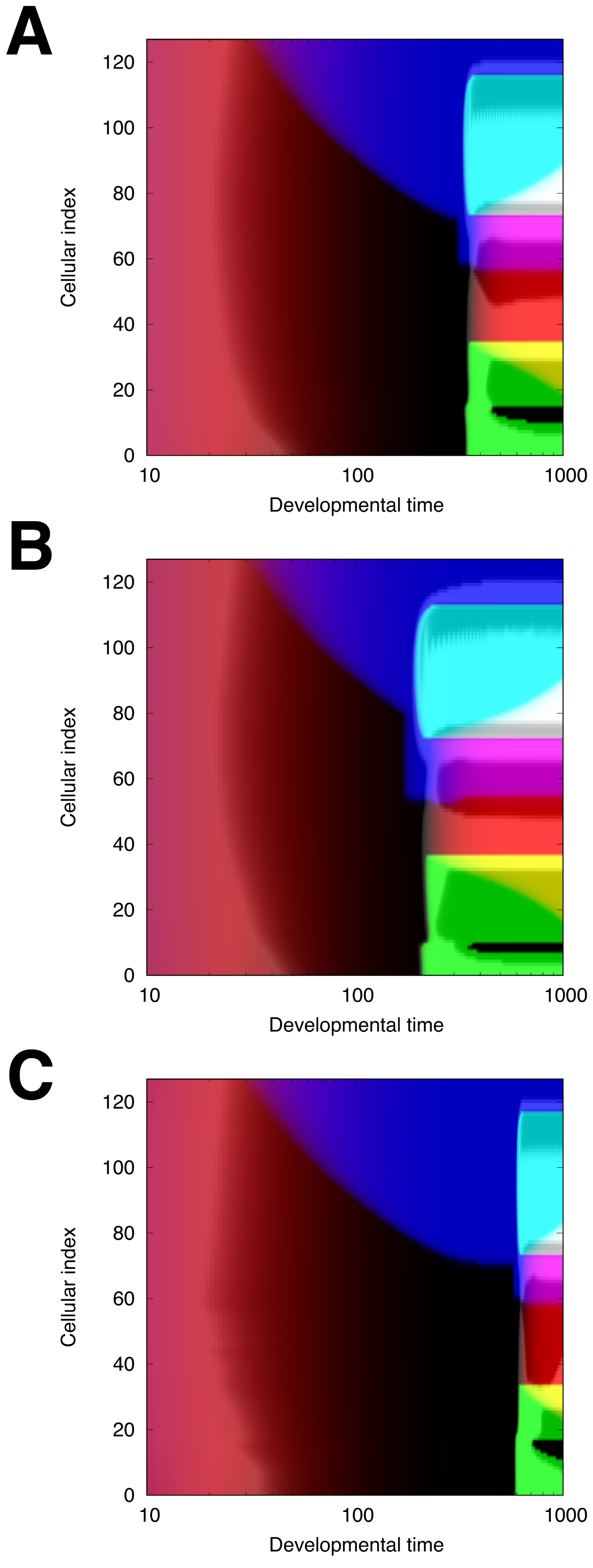

Supplement: S6 Fig — Subfigures A)–C) display pattern formation dynamics of the same gene network except for the change in the time-scale parameter of the slowest gene. A: Pattern formation dynamics originally obtained from evolutionary simulation. The value of the timescale parameter of the slowest gene is c5.17×10−2. B: Pattern formation dynamics with a larger timescale of the slowest gene; 10.3×10−2, 2 times larger than that of A. C: Pattern formation dynamics with a smaller timescale of the slowest gene; 2.58×10^−2^, 0.5 times smaller than that of A. (TIF) [file pcbi.1011867.s006.tif]
