# Supplementary material for: Developmental hourglass: Verification by numerical evolution and elucidation by dynamical-systems theory
Source: PLoS Comput Biol. 2024 Feb 29;20(2):e1011867. doi: 10.1371/journal.pcbi.1011867 (PMC10903806; doi:10.1371/journal.pcbi.1011867)
Supplement: Attachment — Submitted filename: referees_report.pdf [file pcbi.1011867.s007.pdf]

Reviewer's report
Summary:

To explore the origin of the developmental hourglass, the authors developed a simple numerical model consisting of many cells containing gene regulatory networks coupled by diffusion. They conducted evolution simulation numerically. From the population taken from intermediate generations of the initial simulation, they made a clone population and conducted the evolution simulation, which models different species sharing common ancestors. By comparing the species thus generated, they found that the similarity in the gene expression is highest at some intermediate time during development. It corresponds to the developmental hourglass. At that point, many genes changed their expressions drastically, and the cell state changes largely. They found that expressions of a few genes change slowly in the initial stage of development, and thus they are considered to control the developmental process.

The paper provided an important insight into the developmental hourglass using numerical simulations. The results are sound and give some explanations for experimental observations. Thus, this paper is suitable for publication in PLOS Computational Biology. However, some points are not so clearly written. In particular, some of the results and methods need more explanation. I think that the paper needs some revision before publication.

Major points:

1. I feel that the phrase in the title "confirmation by numerical simulation" is too strong. The word "confirmed" is also used in the first sentence of Discussion. As far as I can understand, this work successfully generated an hourglass-like developmental process using a simple model. Although the findings are impressive and important, I feel they are still far from "confirmation" of experimental observations. Some weaker expression will be suitable.

2. The morphogen gradient is important for cell differentiation to occur. However, I could not find any mention of the morphogen gradient until I read the Method section. I think it should be mentioned, at least briefly, before the results are described. Otherwise, readers also may be confused about the origin of cell differentiation. Also, the number of cells, M, is not provided even in Method (I only found it's more than 120 from the Figures).

3. Fig2. A2 is for one individual that has a common ancestor with the individual shown in A1. Other examples from the same simulation (is it right?) are shown in S2F. But there are a hundred individuals in one population (the number of genotypes may be less). Are they all behave similarly? Although the phrase "always in the middle of ..." is used in the main text, it is still not clear if there is no exception. If all the individuals exhibit hourglass-like behavior when the derivative species are close in the phylogenetic tree, it should be clearly stated.

   I feel that the distinction between "examples" and "all" is not taken care of throughout the paper. If all the individuals behave similarly, it should be stated. And if there are exceptions, it also should be mentioned.

4. Seeing Fig.2 and S2F, I found that the progression patterns of the similarity indicator are diverse between individuals. Then, I wonder how it progresses if the similarity indicator is calculated between individuals in the same species. The result will serve as a reference to estimate the extent of similarity between different species.

5. Caption of Fig.3
   The readers cannot judge if the sentence "In this case, for the pair branched at the recent 100-300 generations" is correct, because only the data for "200 generations (blue)" is shown in the corresponding range, and it seems that the green line (500 generations) also exhibits a peak at the same point.

6. The definition of the pleiotropic genes is unclear. Since the individual has many cells with different cell states, I think the gene expression levels differ from cell to cell. In such a situation, how are the pleiotropic genes identified? Some more detailed explanation is required.

7. p.14 second paragraph
   Although the importance of the noise in the initial condition is stressed for the emergence of the developmental hourglass, I could not find how the noise in the initial state is introduced except for the clone experiment in Fig.5.

8. p.14 second paragraph (The discussion on the robustness)
   Although the authors wrote "Hence, during the ...", it is not confirmed in this paper. Thus, this part is not appropriate to be written here. This point should be discussed in Discussion.

9. p.15 the last paragraph
   I could not find a description of how the time scale of the slow genes can be changed.

10. Discussions, 2nd paragraph
    At the end of the second paragraph, the perturbations in the initial condition is mentioned. But I could not find the description of this perturbation.

11. Discussions, 6th paragraph
    In this paper, the term "species" and "phylum" are used rather arbitrarily in discussing the results. It is not clear how we can define "species" and "phylum" in this model. In this connection, the last sentence "This result corresponds..." seems to be sloppy.

12. Discussions, 7th paragraph.
    The meaning of the sentence "when such processes..." is unclear. Since the cell interactions described in the former sentence are not investigated in this paper, more reasoning should be written for discussing them.

13. Methods: Gene expression dynamics
    I think r.h.s. of equation (1) may be

    $$F(i, \boldsymbol{x}(r,t)) - \gamma_i x_i(r,t) + D_i \ldots$$

    In eq.(1), the diffusion is expressed by the spatial second derivative. But in this model, the cell positions are discrete, and the spatial distribution of x_i within cells is not considered. Thus the diffusion effect should be described by the difference between neighboring cells rather than the spatial derivative.

14. Methods: Initial/boundary conditions
    I could not understand the meaning of the sentence "the expression levels of each gene...".

15. Methods: Fitness function
    ON and OFF are defined as > 0.99 and < 0.01, respectively. Then, there should be the intermediate range of the expression. Seeing Fig 6B, expressions of some genes at the final state take intermediate values. If the final states of the target four genes are always either ON or OFF without exception, it should be mentioned. In that case, the

mechanism for target genes to reach either an ON or OFF state should be described briefly. In case some exceptions have been observed, they should also be mentioned.

16. Methods: Mutation
The list of evolvable parameters is lacking.

Minor points:

1. Fig 2C and 3
The title of the vertical axis should be "similarity indicator".

2. Fig.5
The definition of the total variance in gene expressions is unclear.

3. P.14 second paragraph
Fig. 4 may be Fig. 5

4. Fig.6
The definition of the variance in the timescale in C is not clear. Are these variances calculated for timescales over different cells?

5. Methods: Calculation of variance...
x_l should be x_i. At the end of the paragraph, there is a summation over j, but I think it should be the summation over i.
