# Supplementary material for: Developmental hourglass: Verification by numerical evolution and elucidation by dynamical-systems theory
Source: PLoS Comput Biol. 2024 Feb 29;20(2):e1011867. doi: 10.1371/journal.pcbi.1011867 (PMC10903806; doi:10.1371/journal.pcbi.1011867)
Supplement: Attachment — Submitted filename: Response_to_Reviewers.pdf [file pcbi.1011867.s008.pdf]

Reviewer's Responses to Questions
Comments to the Authors:
Reviewer #1: Kohsokabe et al. present an unusual approach to provide some mechanistic insight into the hourglass model of morphological evolution through simulation. They present an admittedly simple simulation framework that implements simple gene regulatory networks in 1D organisms with cell intrinsic and cell signaling effects. They simulate their ontogeny and evolve them over 1000 generations. Species are periodically split off from the simulated organisms to provide comparative data. Noisy initial conditions and mutations in GRN matrices at species transitions are implemented. Cell type diversity (under simplified, but strict definition) is used for selection of individuals between generation. They then develop a framework to compare spatial gene expression patterns that evolve in this simulated system across developmental time. This provides the ingredients for testing the predictions of the hourglass model. They find that indeed, in this system, mid stages of the developmental trajectory are more conserved between species compared to earlier and later stages, mid stages are enriched for pleiotropic genes (under the definition of the toy model) and show less divergence within species evolved from the same starting conditions (with noise). All this seems to mimic the general predictions of the hourglass model. Detailed analysis of the evolving GRNs indicate that the pattern could be driven by relatively few genes that change their expression slower relative to other genes. This is interpreted in the context of the theoretical work previously published by the authors on the subject.

Thank you for your positive evaluation of our manuscript and constructive comments. We really appreciate your insightful comments.
We have revised the manuscript following your comments. We have expanded the explanation of the Model, and the description of the results by adding and revising several figures and supplemental figures and expanded the discussion. Point-by-point replies are attached below, while the revised texts are highlighted in yellow. We hope that the manuscript is now acceptable for publication.

1. **The assumptions of the simulation seem reasonable. It is fairly difficult to understand the Figure 2 visualization of the two species and how they arose, however, once the distinction between ontogeny (developmental time, plotted) and phylogeny (the number of generations that led to this individual**

and potential split along the path) sinks in, the paper is relatively easy to follow. The hourglass pattern is much stronger for more related species and all but disappears when the bifurcation appears early in the simulation. This is not necessarily the prediction of the hourglass model. The authors should discuss this.

Thank you for your positive evaluation of the manuscript. We agree that the gradual disappearance of the peak as the branching time increases is not necessarily observed in the hourglass model, but the comparison across species may not be sufficient to disprove the gradual change. Furthermore, we found that the disappearance of the peak in our model is not necessarily so gradual when we have examined the peak more carefully. As shown in the later section, the similarity peak is discernible, when the two branched species share the common slow genes that mainly control the expression. In the real data, the range within which the common hourglass behavior is observed is not fully understood. Some suggest that the hourglass is seen in the common phylum, whereas future studies will be required. One possibility will be to connect the common slow genes (or slow control process in development) with the phylum, but at the moment this is just one possibility. We have relaxed the argument of this point and discussed the above point.

We have added a remark on this point at the 6th paragraph of the Discussion as follows.

Although the decrease in the peak might look gradual, but at the point when the commonality of slow genes is lost, the peak disappears distinctively. Here, the attenuation of initial variation should always be present, the species separated earlier would use different gene sets for the slow process, and then the gene expression patterns of the two species would no longer be similar in the intermediate developmental stage, and the similarity indicator peak would no longer exist. In this case, the similarity can be maximal either in the very beginning or at the very last developmental time (Fig 3). This result may correspond with the cross-phylum comparison of gene expression patterns, as reported previously [41] (whereas we note the criticism to it as discussed below). It will be important to examine closely within which range the common hourglass behavior is observed.

2. **Also, given the obvious distance between this simple toy model and real**

evolving organisms, the authors should think about other possible, perhaps more trivial reasons for why the mid-simulation pattern convergence occurs.
Please note that we have not included any fitness condition that favors the hourglass. Furthermore, the bottleneck is not observed, if the amplitude of initial noise is low, which suggests that the noise reduction process is important for the appearance of the hourglass. In fact, at the bottleneck time, variance over different initial conditions (over the clones) takes a minimum. Also, if the two species do not share the common slow genes (process), the similarity peak is not observed. Further, by changing the timescale of this slow process, the bottleneck time changes accordingly (Fig.6). These suggest that the present interpretation of the hourglass is valid, and the hourglass we observed is not due to some trivial artifact of the present model. We have added a remark in discussion.
We have added a remark in the 2nd paragraph of the Discussion and also expanded the discussion on slow genes (8th paragraph)

3. **The gradual decrease in gene expression noise in the system is intriguing. The sudden rise of complexity in the system after that would require more thorough investigation.**
As briefly remarked already in the previous manuscript, the sudden rise in the variance could be due to a slight difference in the timing of the onset of slow gene expression, which causes a large variance. In fact, the increase after the bottleneck is always observed whereas the sudden rise to make a peak is not generally observed. In most examples, the increase of variance is rather gradual. Here, we have updated Fig.5 so that several examples are plotted and added a remark on this point. We have added the following sentences, after the explanation of Fig.5. as follows:

After the variance takes minimal at the hourglass bottleneck time, it increases, as shown in Fig.5. In most cases the increase is rather gradual towards the final stage of development, whereas a peak is observed in some samples. This is due to a slight difference in the timing of the onset of the rise in the slow gene expression (as will be discussed below), which causes enhancement of the variance in some gene expression.

4. **The authors make the connection with slow genes. However, this idea is not sufficiently developed. Would there be a way to investigate why does this**

dynamics coupling occur in this system? And if yes, could there be drawn some analogy with GRNs in real biological systems?

We have added a new figure of GRN to show that the dynamics of the slow genes control the dynamics of the genes downstream. Working subnetworks go downstream originating in the slow genes. (Similar observations are also observed in our earlier paper [38], discussing the evolution-development congruence). We have added a new figure, figure 7, to show this change in working subnetworks originated in the slow genes and added an explanation.

5. **Overall, this is an unorthodox view of the hourglass model. Understanding the toy model and looking at its behaviour could be of interest to scientists who are in general struggling with the meaning, significance and mechanisms of this peculiar embryological observation.**

   Thank you for your evaluation. As the understanding of developmental hourglass has not yet been established, we hope the present study will contribute to it by shedding a new light from the perspectives of robustness to noise and dynamical systems.

6. **Minor comments: The paper contains several typos. Figure 1: developmetnal time 2x,**

   We assume you mean the typos in Fig.2.

   Thank you for pointing these out. We have corrected developmetnal to developmental.

7. **Figure 4 is not referred to at all, the relevant call out goes to Figure 3 again.**

   We have corrected the typos, and referred to Fig.4 on page 4

8. **Fonts in Figures are very diversified, different fonts and sizes.**

   The fonts are identical, but for figures including subfigures, they may look smaller. Also, when the manuscript is published, the size of the figures will be adjusted so that the fonts and sizes will be coherent. In case the size is too small, we have enlarged the size of the font to increase the visibility.

Reviewer #2: Phenotypic evolution has been driven not only external factors such as environmental conditions, but also by internal ones, i.e., developmental processes. One of the key interests in evolutionary developmental biology lies in

deciphering the evolutionary constraints and principles imposed by embryogenesis. The paper under review tackles this intricate issue by utilizing evolutionary simulations, a noteworthy methodological choice that distinguishes this study within the evo-devo field. This approach allows the authors to gain a new valuable insight into a problem that has been traditionally addressed by speculation based on observations of extant species and fossil records.

However, the paper presents several significant concerns. The most prominent point is the ambiguity in the presentation of the methods, data interpretation, and the validation of their arguments. These shortcomings necessitate a major revision of the manuscript. The severity of these issues is compounded by errors in crucial equations explaining the model. Coupled with the lack of explanation for some variables and functions, the paper in its current state poses challenges for precise interpretation. The paper would be greatly improved by revising its data presentation and argument validation, which ensure credible conclusion.

Thank you for your positive evaluation of our manuscript and constructive comments. We really appreciate your insightful comments.

We have revised the manuscript following your comments. We have expanded the explanation of the Model, and the description of the results by adding and revising several figures and supplemental figures and expanded the discussion. Point-by-point replies are attached below, while the revised texts are highlighted in yellow. We hope that the manuscript is now acceptable for publication.

For "Model":

1. **The manuscript currently lacks information for cell count and cell proliferation. It will help understanding if the authors incorporate explanations about these factors.**

   We have added a text to explain that "An individual consists of 100 cells allied in a one-dimensional space with no cell proliferation during the developmental gene expression dynamics, and also added a further description of the model".

For "Gene expression dynamics":

2. **Equation (1) appears to be missing a ")" which compromises its correctness.**

   We have corrected the typos.

3 To enhance comprehension of the model, the authors should provide precise information about several key elements.

3.1 Equation (1) seems to describe changes in protein expression, but it remains unclear how generation is accounted for.

3.2 D_i is assumed to be a value related to diffusion, but without explanation, its exact role and method of determination remain ambiguous. This lack of clarity extends to the setting of cell-cell interactions.

3.3 The functions f(x) and F(i,x(r,t)) need proper descriptions, as their roles are currently unexplained.

Following your suggestions, we have thoroughly revised the Methods section. We have added some missing information, and explained the setting more carefully.

3.1: Please note that we omitted the index for generation here, as too many superfix/suffix is not kind to readers, even though the matrix J_{ij} is changed by generations. The equation (1) is for J_{ij} for given generation, i.e., $J_{ij}(k)$ for generation *k*. For simplicity the index *k* is omitted. We have explained this in the revised manuscript. We have added the following sentence after eq.(2) as follows.

"In the above equation, J_{ij} changes by generation k through evolution, but putting the index k here makes the expression hard to see, so it is omitted here."

3.2: D_i is the diffusion of component between neighboring cells as in Eq.(1). This gives cell-cell communication, and allows for gradual change of the concentration along the space. We have added this explanation. We have added the following sentences:

"The second term in equation (1), $D_i\big(x_i(l+1,t) - 2x_i(l,t) + x_i(l-1,t)\big)$ is a discrete-space version of the diffusion, which denotes cell-to-cell interaction via diffusion where D_i is the diffusion constant of the product of the gene i, that determines the rate of diffusion."

3.3: We have added the description and meaning of F (and f) with care.

4 The correspondence between "as x is increased" and "as x_i (l,t) decreases" needs to be clarified.

We apologize the typo. The expression x need to be replaced by $x_i(l, t)$. We have corrected it.

For "Initial/boundary conditions":

5 **Explanations are required to better understand this part of the manuscript**

5.1 **It is assumed that M represents cell numbers, but without explicit clarification, this remains uncertain.**

We have replaced M by L and also revised the description.

5.2 **The authors claim that gene expression levels are "spatially uniform" for genes other than gene #0 and 1. It is unclear whether this signifies identical gene expression levels across all cells.**

Here, we meant that the expressions of other genes are spatially uniform whose values are different by genes. The previous description was unclear, so we have revised the description thoroughly.

5.3 **inheritance of gene expression levels from ancestors needs further explanation. Does it imply that the clone inherits the initial state used by the progenitor cell?**

To make the meaning clearer, we have revised the description.

For "Selection":

6 **More information should be given about the variable e^(f_i) to clarify its role and purpose.**

In general, f_i or any increasing function of fi could work for the present study. However, to make the evolution process faster, it will be appropriate to adopt the form that the increase in the number of cell types effectively influences the increase in fitness. For it, we have introduced the exponential function (in fact, such an exponential form is often adopted in genetic algorithms or numerical evolution). We have added a remark on this point in the Selection part.

For "Mutation":

7 **There appears to be no list detailing the evolvable parameters and their potential changes. Provision of this information would assist readers in comprehending the model.**

We added a table to describe basic parameters, distinguishing evolvable and fixed parameters.

For "Calculation of the similarity indicator of gene expression patterns":

8 **The variable σ seems to be absent in the final sentence.**

We have corrected it.

For "Calibration of the timescale of slow gene":

9. **There is ambiguity on the definition of terms and the purpose of calculations, which should be resolved.**
10. **The concept of calibrating the timescale needs an explicit explanation. The authors should provide a detailed definition of the timescale, clarify the purpose of its calibration, and justify the employed manipulations.**
11. **The relationship between the introduced value k and the term "timescale" remains obscure.**

We apologize that the explanation of calibration is hard to understand. The timescale is determined as follows:

Consider time varying function y(t) which, in the present case is x(r,t). First take a time span in which y(t) monotonically changes (i.e., its derivative does not change the sign). The time scale Td is roughly given by the Δt, in which the difference |y(t+Δt)-y(t) | exceeds some threshold, i.e., the timescale is the time span in which this difference goes beyond given threshold δ: if the change is faster, Td is smaller, roughly speaking, the time for a gene to reach saturated expression level. Now to implement this idea, we set small Δt, which is the time interval for numerical simulation and take kΔt with increasing k as 1,2,3,.. and obtain maximal k such that it satisfies |y(kΔt)-y((k-1) Δt) | <δ. Then the time scale Td is given by kdΔt. In the numerical procedure, we take δ=0.001 and Δt=1, whereas the result is invariant as long as δ is 0.0005~0.005.

We have added this explanation at the "Estimate of the timescale subsection in the Method section.

For paragraph 1:

12. **The term "fixed terminal state" requires explicit definition to avoid ambiguity. The Methods section mentions that fitness is determined by the number of final differentiation states, which led me to an interpretation of no specific target expression pattern. clear definitions are necessary to grasp the simulation setup accurately, namely whether this understanding is incorrect (there is some target pattern), or "fixed state" refers to a "steady state".**

We agree that "fixed terminal state" is misleading. We used the term borrowing

from the fixed-point in the dynamical systems, but as there are no fixed terminal states. We have revised it to the steady state. Also, following your comments, we revised the definition of the fitness function. We have also added a remark on the case where individuals with oscillatory or chaotic states are excluded.

13. **Gene expression patterns appear to be changing even at the 1000th timestep, as inferred from Fig 2A. The reason behind the choice of ending "embryogenesis" at the 1000th step requires clarification.**
    Due to the emergence of slow processes, it takes a bit more time for the state to reach the final steady state. We have made a longer simulation. In this specific example, there remains some oscillatory gene expression at some localized space, whereas the existence of the maximal (16) cell types is assured by the existence of fixed cell types at other regions, so that the maximal fitness is achieved. Such localized oscillation is not essential to the present hourglass argument. In fact, for many other examples, the final state is a completely fixed-point. We have commented on this point in the caption of Fig.2A .
    We have added the above point in the caption of Fig2A.

For paragraph 2:

14. **The legend for Fig. 2A states that "Spatial pattern transitions were more frequent in the early developmental time." However, spatial differentiation seems minimal until around the 100th step. It would be beneficial if the term "spatial pattern transition" is clearly defined, quantified, and compared across developmental periods.**
    Here, the spatial pattern change means changes in the on/off expression of genes over the space. If the expression of genes shows on/off change in some spatial region (i.e., non-uniformly in space), this means the spatial pattern changes. In the two-dimensional color plot for space and time, this is seen as a generation of stripes (or domains) in which the expression of (target) genes is distinct from another spatial region. Up to 100 time steps, such changes are infrequent.
    We have added this explanation in the caption of Fig.2A.

For paragraph 3:

15. **The statement "there are some phases within which patterns change only slightly" needs clarification whether it refers to subtle changes in ontogeny or phylogeny.**

We meant changes in ontogeny. We have revised the preceding statement: “By examining the ontogenetic change of spatial patterns with the differentiation of cell types, it was noted that there are some phases within which patterns change only slightly in ontogeny”.

16. **In the legend of Fig. 2B, the "middle" period is suggested as the stage of highest similarity among lineages. If it refers to the 100th step out of 1000, it seems to fall within the "early" period, thus requiring a precise definition of the "middle" period. While I comprehend that the term "middle" does not necessarily imply a literal midpoint, and the primary focus here is the hourglass-like pattern, the current manuscript would lead to confusion for readers.**
    Yes, the term middle might be misleading. The point we want to make here is the high similarity exists neither in the beginning nor at the end of the development. We revised the statement as “Note that the high similarity region is approximately 100-200 in either individual developmental time, which is intermediate of the whole developmental dynamics.”
    We have adopted the term "intermediate" instead of “middle” throughout the manuscript.

17. **The previous observation of the phylotypic period suggests that the conservation of this period is persistent, regardless of the evolutionary scale. Thus, if the authors claim to recapitulate an hourglass pattern in their simulation, persistent conservation at a specific period must be demonstrated.**
    By comparing the species evolved across different generations, and examining if they share the common bottleneck period, your question will be addressed. We have examined a few examples; for instance, in supplementally figure SF4, we compared the species at the 1000th generation and every 100 generations distant ancestor (not the comparison between species evolved from every 100 generations distant ancestors as in SF3). They show the common similarity peak as that presented in Fig.3 and SF3. After the fitness is maximal by the evolution, the common slow mechanism (as is discussed later) is preserved, and changes occur only at the later stage, so that the intermediate developmental stage will be preserved. To completely answer your question, the comparison across species of all different generations may be necessary, which, however, demands too much

computation, and is beyond the scope of the present study. We hope that the above example will be suggestive.

We have added a new Supplement Figure SF4 and discussed the above point. We have also added the following text, prior to Fig.3

Note that the common similarity peak is also observed even between the evolved individuals and their ancestors, for instance, seen in Supplementary Fig.4, where we compared the species at the 1000th generation and its ancestor at the 500th generation. This will be because the mechanism leading to the similarity in the intermediate developmental stage, as will be discussed later, has been conserved after the acquisition and will be preserved after maximal fitness is achieved by evolution.

18. **Regarding Fig S1, there seems to be a mislabel on the y-axis.**
    We apologize the typo. We have corrected it.

19. **For Fig S2, details on the number of evolutionary trials conducted, and on the probability of the hourglass-like evolutionary outcomes would enhance the paper's value.**
    We have carried out 21 runs for a set of evolution simulations of the present model, where different spatial patterns are observed after reaching the fittest state. Among 18 of 21, we have observed the present hourglass. (In fact, the exceptional 3/21 cases in which a clear intermediate peak was not observed, contain oscillatory gene expression in the earlier developmental stage, which masks the peak) .
    We have added the above statistical results in the text (page 11).

For paragraph 4:

20. **The authors claim that the "middle" period was consistently conserved between two species that diverged at the 800th generation based on one example. This assertion would be greatly strengthened by the addition of statistical data, such as the distribution of the most conserved period. This information would be particularly beneficial when discussing whether the "middle" conservation observed in this simulation persists like the phylotypic period.**
    As replied above, we have added the statistical data, showing that the similarity

increase peak in the intermediate stage was observed for 18 of 21 runs.

For paragraph 6:

21. **In Fig. 3, the authors compared cellular expression patterns between lineages representing extant species. It would be helpful to understand the similarity of developmental patterns between ancestral species (e.g., species of the 400th generation that diverged at Branch C). If the mid-conserved period in this simulation indeed represents the phylotypic period, it is expected that ancestral species also exhibit mid-phase conservation. Comparing ancestral species would also shed light on the process of establishing constraints on the conserved period.**

    After the highest fitness and slow-gene mechanism are gained, the similarity peak is observed, even for ancestral species. Please see the reply to 17 and the corresponding supplementary figure.

22. **The findings from Fig S3 reveal diminished conservation of the mid-phase between "distantly" related species (including those 300 generations apart within the 1000-generation simulation). This might indicate that the simulation's scope extends beyond the within-phyla evolutionary scale, which is traditionally where the hourglass model is applicable. The authors should address the appropriateness of the simulation's scale.**

    As seen in the Supplementary figure SF3, the disappearance of the peak might look gradual as the branching time increases. However, the disappearance is not necessarily so gradual when we have examined the peak more carefully. As shown in the later section on the slow gene, the similarity peak is discernible when the two branched species share the common slow genes that mainly control the expression of other genes.

    In real biological data, the range within which the common hourglass behavior is observed is not fully understood. Some suggest that the hourglass is seen in the common phylum, but it is still controversial and future studies will be required. In the present study, the range sharing the peak is discussed later in connection with the range sharing the slow gene (or slow control process in development). We have added some explanation on the range revised the 6 and 7th paragraphs in the discussion, and relaxed the argument to connect the slow gene with the phylum.

23. **The observed pattern that developmental similarity peaks at the final developmental stage among species separated by over 300 generations may imply developmental drift, as opposed to developmental constraints. The authors are recommended to share their interpretation of this observation.**
We assume that you mean the small peak at around 300 time steps (not generations) in Fig2. This small second peak would be just due to the difference in the timing of the expression of downstream genes from the slow ones. In fact, the existence of such second peak depends on the case, and for other cases, it does not necessarily exist. It might be interesting to relate it with developmental drift, but we think the present study does not have the resolution to discuss such subtle process. We have added a remark in the caption of Fig.2C.

paragraph 7:

24. **I encourage the authors to clarify how many evolutionary trials were executed, and within those, how frequently the results exhibited an hourglasslike pattern, and how often they demonstrated enrichment of pleiotropic genes during the conserved middle period. Statistical data on these aspects would be useful in evaluating the validity of the findings.**
As replied already, we have added the statistical data on the observation of developmental hourglass.

For paragraph 8:

25. **During actual embryogenesis, developmental noise is continuously generated. However, in this simulation, noise is only introduced at the initial step. It's unclear if this methodology accurately represents real embryogenesis and requires an additional discussion.**
We have also carried out the simulation to put the noise not only at the initial condition but throughout the developmental time. In fact, we also computed the variance among individuals (of the same network) by adding continuously the noise in the developmental process. In this case, we again observed that the variance to noise is minimal at around the bottleneck time (please see the figure below). Anyway, the noise at the initial stage matters more than that at the later time. We have added a remark on this point on p11 as follows:
"Furthermore, we also computed the variance among individuals of the same network, due to continuously added noise through the developmental process. In this case again, we observed that the variance due to the noise is minimal at

around the bottleneck time."

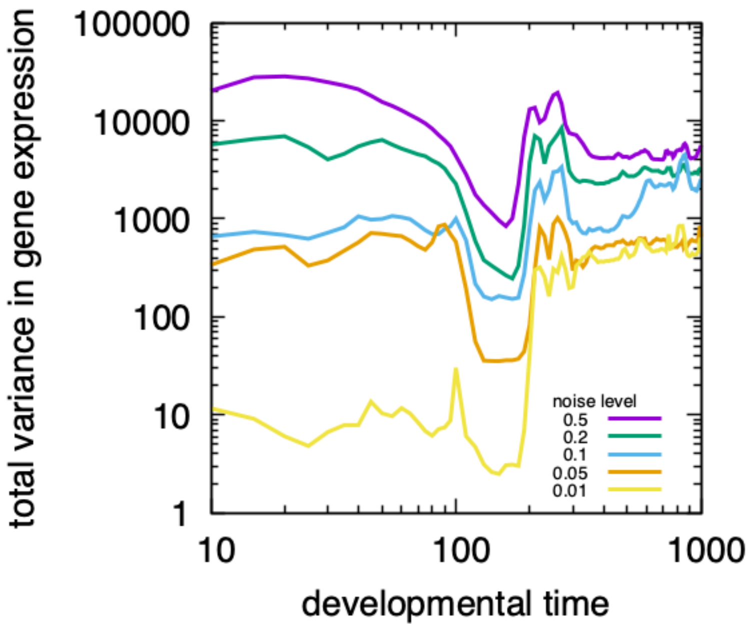

26. **The manuscript says Fig 4 displays the variation among clones, but it appears this is represented in Fig 5.**

    Thank you for pointing this out. We have corrected it.

27. **The indicator for gene expression pattern similarity in this analysis is different from those utilized in other assessments of similarity. An explanation for this deviation would be helpful.**

    In the simulation, we can get all the data for all the expression levels over cells over developmental time, so that this choice of index will be relevant, as it utilizes all the information, including the spatial information that is not easily available in the experimental data analysis. Still, we agree that the index most experimentalists adopt is different. So, we have also computed the index that the experimentalists often adopt. As shown in SF1, the results are essentially the same; we observed the peak of similarity at around the same time of development. We remarked on this and added a new Supplementary Fig.1.

For paragraph 9:

28. **The statement referring to the necessity for developmental noise to achieve robustness requires further specificity. It should clearly define the context of robustness - against what and of what? Furthermore, the corresponding**

setup within the simulation that reflects this requirement needs explicit clarification. There seems to be a disconnect with the setup that aims an increase in the terminal differentiated state throughout the simulation. Given this ambiguity, the rationale behind the attenuation of initial noise is not clear.

First, we apologize for the description of how the noise on the initial condition is numerically implemented, which was missing in the previous manuscript. We added a detailed description of that in the Initial/Boundary condition subsection in the Methods section.

This said, the rationale behind the importance of noise lies in recent studies on the relevance of robustness against noise to evolution, as well as to mutational robustness. As recent experimental studies elucidated, gene expression dynamics are rather noisy, so the protein or mRNA abundances vary due to such chemical-reaction noise. (see e.g., Elowitz M.B., et al. (2002) Science, 297(5584):1183) For fitted cells or organisms, robustness to noise is essential; otherwise, such fitted states are not sustained by noise. In development, such noise exists from the maternal environment, and the noise effect exists from the earlier stage, as there is a relatively large variance in the egg (see Ref 26 by Newman). As the developmental process is complex and involves many steps, achieving robustness of adult organisms (or fitness therein) to noise, in particular, that to earlier stages is essential. Hence, it is naturally expected that the phenotypic variance by noise has to be reduced, at least up to some developmental stage. In fact, the present simulations suggest such a reduction. Of course, we agree that further experimental confirmation of such a reduction of variance is needed in the future. Also, the picture of the evolution of robustness to noise is consistent with the stabilizing selection that has been discussed over decades in evolutionary studies (Schmalhausen, I. I. (1949). Factors of evolution: the theory of stabilizing selection).

We have added a remark on this at the 3rd paragraph of the discussion.

For paragraph 12:

29. A clear description of what specifically is changing in a stepwise manner in gene expression patterns would enhance understanding. Providing a quantitative evaluation, or conversely, an example of gradual change, would be beneficial.

As the gene expression dynamics involve a reaction process with a high Hill

coefficient, as represented by the sigmoidal function given by F(x), stepwise change of gene expression between on and off is often observed unless the input to each gene remains a value near the threshold in F(x). For instance, the range of input to show a gradual change is approximately within the 1/β, with β as Hill's coefficient in the model. Given that is 40 throughout the simulations, the input range for the gradual change is so small that the expressions of most genes change in a fast, stepwise manner. (Exceptions are the change in expressions of slow genes, which are comparatively smaller than other genes, to suppress the speed of gene expression change.)

This explains stepwise expression change in time. Stepwise change in spatial pattern indicates that gene expression level changes between on and off, as a result of expression changes throughout the space. To address this, we added Figure 7, which shows an effective substantial gene network and gene expression dynamics and illustrates how the stepwise change in pattern was controlled.

We have added a corresponding explanation to the paragraph.

Paragraph 13:

30. To effectively argue that a switch occurs between initially expressed genes and late-expressed genes around the bottleneck period, the authors should support their findings with statistical analysis, as opposed to merely providing an example.

As in the previous reply, we have added statistical data.

31. Ref [23] investigated developmental conservation in terms of temporal variability of gene expression patterns. Their analysis highlighted larger temporal expression divergence before, rather than after, the bottleneck period. A discussion on the compatibility between the authors' findings and those from this previous study would be appreciated.

The main claim in [23] is that the variation of gene expression across mutants is minimal at around the bottleneck. As you pointed out, the variation is larger before rather than after. Please note that the on/off switching is more frequent after the bottleneck in their result. This is just the temporal variation, and it does not necessarily mean that the variance across mutants (or by noise over isogenic individuals) is much larger at that stage. As shown in Fig.5, the variance of expression levels across initial conditions (and also over mutants) is large in the initial stage, and then decreases at the bottleneck, and increases at the final

stage. Also, right after the bottleneck, there occurs frequent on/off changes in gene expressions. In this sense, our result and that in [23] is not inconsistent. Whether the variance at the initial or final stage is larger depends on the choice of initial noise level and details in the model, so the comparison is beyond the scope of the present study.

Paragraph 14:

32. **In Fig 6b, the rate of gene expression change is gradual, making it difficult to classify genes as "slow", "normal" or "fast". The authors should define a clear and quantitative indicator.**
    As in Fig6B, changes of some genes require about 100 time steps in the model, whereas most others change within the time scale less than 10 steps (see also newly added Fig.7). This is commonly observed as a result of the evolution of the present model. Here, we use a criterion for the slow gene that has a time scale larger than 100. On the other hand, in the network structure, as shown in the newly added Figure 7, the slow genes influence other faster genes.
    We have added this description to the paragraph.

33. **The authors argue that numerous genes are regulated by slow genes. They should clarify whether this is based on solid evidence or if it is speculative**
    We have added new Figure 7, which describes the structure of a gene regulation network to show how slow genes control faster ones and added an explanation for it.

Paragraph 15:

34. **Fig 6d illustrates the correlation between the timescale of slow genes and the similarity peak. However, in substantial instances, the slow genes reach their expression change peaks after the similarity peak (provided my understanding of the "timescale" of genes is accurate). This may suggest that the data presented here doesn't fully support the causality between slow genes and developmental conservation. The authors should discuss this point.**
    First, the timescale here is not the timing of the expression change but is the inverse of the speed of gene expression change and is computed by the absolute value of the time derivative of gene expression dynamics. To avoid the overestimation of the timescale, we stopped counting the timesteps after the absolute value was below the threshold (which is 0.001; see also Methods).

In Fig.6d, the slow genes do not reach the minimum level, but still, the change started earlier, so the influence has occurred already, even though drawing a conclusion for the causality may be difficult. Still, as seen in the newly added Figure 7 for the gene network structure, one can see the influence from the slow genes to the target genes. We have commented on this.

35. **The color codes in the figure legend appear to be misaligned.**
    We have corrected the error.

For paragraph 2:

36. **The simulation implies that substantial developmental noise is essential during evolution. Is there empirical evidence that can corroborate this assumption?**
    An increase in robustness to noise or environmental variation through evolution has been discussed over decades as stabilizing selection since the pioneering argument by Shmalhausen. Such evolution of robustness to noise is natural, as the developmental process involves stochastic gene expression, whereas molecular evidence for such stabilizing selection has been discussed, including the role of heat shock protein and so forth.
    We have added a remark on this point in the 2nd paragraph of the discussion.

For paragraph 3:

37. **The authors state that a decrease in variance due to developmental noise will lead to a reduction in variance due to genetic difference. The underlying mechanisms of this assumption remain unclear to me (Intuitively, it seems to be natural to conclude that robustness against noise would be a byproduct of robustness against genetic variations). The readability of this section could be significantly enhanced by providing further clarification on the authors' assumptions regarding "robustness."**
    Recent simulation studies of the evolution of gene expression dynamics show that robustness to noise also fosters the robustness to mutation. In the data analysis in the evolution, of course, it is not easy to conclude which is the case, and what is observed will be the correlation. Still, it is also interesting to note that the picture that mutation is the cause has been challenged by Conrad Waddington who coined the term genetic assimilation, which has been suggested to be valid in some cases.

At the moment, our study cannot necessarily dispute the mutation-cause picture, but at least such picture itself is challenged, for instance, also by a recent experiment on bacterial evolution (e.g., Horinouchi T, et al. (2015) Phenotypic convergence in bacterial adaptive evolution to ethanol stress. BMC Evol. Biol. 16:802).

We have added a brief remark at the 3rd paragraph in the discussion.

For paragraph 4:

38. **Once again, it is essential that the authors provide explicit and objective criteria for distinguishing "slow" genes.**

    As replied to the earlier comment, we have added the criterion of slow gene.

39. **The link between the expression of slow genes and the buffering of initial variations remains ambiguous. While Figure 6 indicates a correlation between the expression change in certain genes and the peak of similarity, it doesn't necessarily demonstrate a causal relationship. The authors should support their argument with analytical results, or alternatively, they should refine the manuscript to indicate that the connection is hypotherical.**

    The figure 6D shows the correlation rather than causality, as you mentioned. Still, the data in the figure include those obtained by externally changing the time scale of the slow genes. In this case, as a consequence of control of timescale of slow genes, the peak for the developmental bottleneck changes accordingly. (In this case, we change the timescale of a slow gene, and the bottleneck time changes accordingly, and the latter is a result of the simulation of the gene expression dynamics with the parameter of slow genes.) Of course, it is not a ‘proof’ of causality, but this may suggest possible causality.

    We have added this remark at the first paragraph of page 13.

For paragraph 6:

40. **The discussion is intriguing on the diversification of the mid-embryonic period among lineages and the concurrent buffering of variation within a lineage. Apart from the authors' speculation, are there any additional analytical results that shed light on the process of gene repertoire alteration while maintaining slow gene sets? One of the advantages of simulation approach is its ability to trace the evolutionary process, so an analysis of this kind could add significant value to this study.**

In comparing species diverted, say around 500 generations, we have observed that slow genes are preserved, whereas downstream genes are mutated, which makes the final patterns diversify. Within the species branched at 500 generations, the common slow genes are shared. Furthermore, in the new Fig.7, we have shown that the slow genes control the downstream target genes, and the downstream networks can vary by species. We understand that further studies to construct a variety of networks will be necessary to fully confirm the slow-gene-control picture foe the hourglass, which will be a future problem.

41. **I would like to ascertain whether the authors are aware of the criticisms towards the methodology and conclusions of ref [41]. (Hejnol A, Dunn CW. Animal Evolution: Are Phyla Real? Curr Biol. 2016 May 23;26(10):R424-6.)**
We are aware that the developmental hourglass, especially its relationship with phylum, is still controversial, and there are criticisms of it. As we are not working on the data analysis, and the current study concerns simulation studies by evolution models, we cannot judge the validity of the data analysis. We have added a remark that the idea of connecting the developmental hourglass with phyla is a hypothesis and we have weakened the statement to relate the sharing of slow genes with phylum. We have revised the 6th and 8th paragraphs in the discussion with citation to the above paper criticizing the relation between hourglass and phylum.

For paragraph 7:

42. **In justifying their assertion that slow genes regulate many other genes, the authors cite their own previous research. I believe presenting data from the simulation conducted in this study would strengthen their argument.**
As already replied, we have added a new Figure 7 for the gene regulation network, which shows how slow genes control faster genes, and added an explanation to it

43. **The authors' proposal is intriguing to define higher taxa based on the emergence of developmental systems (gene sets) that impose constraints. I suggest that incorporating insights from the simulations about the process of emergence of such gene sets will make this paper more insightful and impactful.**
As mentioned above, once such slow genes are gained and other genes are controlled, the slow gene sets are preserved, as they are essential to produce

diverse cell types. Furthermore, the evolution is facilitated by using the slow genes. In fact, often, some gene regulatory mechanism are preserved across many species. We have made a remark on this point. We have added a comment in the 7th paragraph of the Discussion.

Reviewer #3:

Reviewer's report Summary:
To explore the origin of the developmental hourglass, the authors developed a simple numerical model consisting of many cells containing gene regulatory networks coupled by diffusion. They conducted evolution simulation numerically. From the population taken from intermediate generations of the initial simulation, they made a clone population and conducted the evolution simulation, which models different species sharing common ancestors. By comparing the species thus generated, they found that the similarity in the gene expression is highest at some intermediate time during development. It corresponds to the developmental hourglass. At that point, many genes changed their expressions drastically, and the cell state changes largely. They found that expressions of a few genes change slowly in the initial stage of development, and thus they are considered to control the developmental process.
The paper provided an important insight into the developmental hourglass using numerical simulations. The results are sound and give some explanations for experimental observations. Thus, this paper is suitable for publication in PLOS Computational Biology. However, some points are not so clearly written. In particular, some of the results and methods need more explanation. I think that the paper needs some revision before publication.
Thank you for your positive evaluation of our manuscript and constructive comments. We really appreciate your insightful comments.
We have revised the manuscript following your comments. We have expanded the explanation of the Model, and the description of the results by adding and revising several figures and supplemental figures and expanded the discussion. Point-by-point replies are attached below, while the revised texts are highlighted in yellow. We hope that the manuscript is now acceptable for publication.

Major points:

1. **I feel that the phrase in the title ”confirmation by numerical simulation” is too strong. The word ”confirmed” is also used in the first sentence of Discussion. As far as I can understand, this work successfully generated an hourglass-like developmental process using a simple model. Although the findings are impressive and important, I feel they are still far from “confirmation” of experimental observations. Some weaker expression will be suitable.**

   We have removed the term confirmation, adopted the term ‘consistent with’ in Discussion, and adopted "Verification" in the title instead of "Confirmation".

2. **The morphogen gradient is important for cell differentiation to occur. However, I could not find any mention of the morphogen gradient until I read the Method section. I think it should be mentioned, at least briefly, before the results are described. Otherwise, readers also may be confused about the origin of cell differentiation. Also, the number of cells, M, is not provided even in Method (I only found it’s more than 120 from the Figures).**

   We have mentioned the importance of morphogen gradient explicitly just before the results are shown and described the number of cells (which is 128), and also mentioned it explicitly in the table of non-evolvable parameters in the Method section.

3. **Fig2. A2 is for one individual that has a common ancestor with the individual shown in A1. Other examples from the same simulation (is it right?) are shown in S2F. But there are a hundred individuals in one population (the number of genotypes may be less). Are they all behave similarly? Although the phrase ”always in the middle of ...” is used in the main text, it is still not clear if there is no exception. If all the individuals exhibit hourglass-like behavior when the derivative species are close in the phylogenetic tree, it should be clearly stated. I feel that the distinction between ”examples” and ”all” is not taken care of throughout the paper. If all the individuals behave similarly, it should be stated. And if there are exceptions, it also should be mentioned.**

   Yes, they show similar behavior. On the other hand, not all the runs show the clear developmental hourglass, and there are few exceptions. Now we have added the statistics of the behaviors (following also the suggestion of another

referee). (page 10). We have not used ‘all’, accordingly, by considering the statistics.

4. **Seeing Fig.2 and S2F, I found that the progression patterns of the similarity indicator are diverse between individuals. Then, I wonder how it progresses if the similarity indicator is calculated between individuals in the same species. The result will serve as a reference to estimate the extent of similarity between different species.**

   The patterns are diverse by species (i.e., those with different gene networks), but the timing in similarity peak differs only a little. Even though their networks are different, they share common slow genes, so the peak timing is governed by the timescale of the slow gene, which differs only a little when compared to the ‘species’ that branched recently. As for the method to compute the similarity, the description in the Method might be insufficient, so we have added more on the computational method to compute the similarity in the section. We also added another Supplement figure (SF1), which shows that the same peak is obtained even if we adopt a different indicator for similarity.

   Now, as you mentioned, it is important to discuss the similarity between individuals of the same species (i.e., “clones” in the manuscript). Due to the variation in the initial condition, the expression levels are diverted within such individuals. Here, however, if we just compare the two clones, a relatively large variation exists. Hence, instead of comparing each pair one by one, we computed the variance over 100 clones. This is indeed the variance of individual expressions shown in Fig.5. As discussed therein, the variance takes minimal at the bottleneck time. As also explained in the Discussion, the variance due to noise and that due to genetic variation is correlated, which will provide a possible explanation for the developmental hourglass. Note that even though the similarity between clones and between species are correlated, the former is generally much larger than the latter, and will not be adopted as a background index for the latter.

Caption of Fig.3

5. **The readers cannot judge if the sentence ”In this case, for the pair branched at the recent 100-300 generations” is correct, because only the data for ”200 generations (blue)” is shown in the corresponding range, and it seems that**

the green line (500 generations) also exhibits a peak at the same point.

In this figure only, one we cannot see that it is seen for the pair branched at recent 100-300, and indeed the one branched at 500 generations before also show slight peak. Still by considering other examples we examined (an example is shown in in SF2), it will be reasonable to say the peaks exist for the pair branched at around 100-300 generations. We have revised the caption as follows: in this example the peak exists up to ~500, whereas including other examples, the peaks exist for the pairs branched at the recent 100-300 generations.

6. **The definition of the pleiotropic genes is unclear. Since the individual has many cells with different cell states, I think the gene expression levels differ from cell to cell. In such a situation, how are the pleiotropic genes identified? Some more detailed explanation is required.**

   Thank you for your comment. Firstly, we follow the definition of pleiotropic genes in ref [35], which may differ from the ordinary use in the field, but it gives a clear prescription. There, pleiotropic genes refer to the genes that are expressed over half of the total embryonic stages. Here, we measured gene expression level over all cells, and computed the averaged expression of genes. Then we determined 16 staging time points and checked if each average expression of gene is larger than a threshold or not. If a gene's average expression is largerr than the threshold in 8 out of 16 staging time points, the gene is determined as a pleiotropic gene. The staging is one of the arbitrary factors, so we adopted the time points with either arithmetically or logarithmically even intervals. This method approximately agrees with the method adopted in ref [35]. We explained the scheme we adopted to identify pleiotropic genes in the Methods section.

p.14 second paragraph

7. **Although the importance of the noise in the initial condition is stressed for the emergence of the developmental hourglass, I could not find how the noise in the initial state is introduced except for the clone experiment in Fig.5.**

   As you pointed out, an explanation for the perturbations in the initial condition was missing. It is given as the variation of x(i) at the initial condition. We have added the following explanation in the Initial/Boundary Condition part of the Method Section as:

   For genes other than the input genes #0 and #1 (i.e. genes #2, #3,...,#N), the initial expression levels of each gene (at t=0) were given spatially uniform. The initial

value is chosen as

$$x_i(l,0) = x_i^{init} + \delta i$$

where

x_i^{init} is a constant value inherited from the ancestor which is slightly altered by mutations (as described later), whereas δ_i is the deviation due to the epigenetic perturbation, given by a random value from the Gaussian distribution with a mean 0 and the variance σ, independently generated for each gene, but taken uniformly over all cells. This noise term is due to environmental, behavioral, and other non-genetic effects, the initial condition is deviated from the inherited value x_i^{init}. Except for the SF4, σ is set to 0.1.

8. **p.14 second paragraph (The discussion on the robustness) Although the authors wrote "Hence, during the ...", it is not confirmed in this paper. Thus, this part is not appropriate to be written here. This point should be discussed in Discussion.**
   We have moved this sentence to the third paragraph in the Discussion and stated it as speculation by following your suggestion.

p.15 the last paragraph

9. **I could not find a description of how the time scale of the slow genes can be changed.**
   We changed the parameter for the corresponding gene so that its time scale is changed. We have added an explanation in the manuscript.

Discussions, 2nd paragraph

10. **At the end of the second paragraph, the perturbations in the initial condition is mentioned. But I could not find the description of this perturbation.**
    As you pointed out, an explanation for the perturbations in the initial condition was missing. First of all, we have added the explanation in the Method Section on Initial/Boundary conditions (see the reply to 7). In this sentence in the discussion, we mean that if there is a sufficient level of variance in the initial condition, as adopted in our simulation, the robustness against the initial noise evolves and the developmental hourglass is observed, whereas if it is set to too little, the developmental hourglass was not observed as in SF4.
    We have also revised the discussion as：
    In fact, when there is a sufficient level of the variance in the initial condition, as

adopted in our simulation, the robustness to the variation evolves, and in this case, the developmental hourglass is observed, whereas if it is set to too small, the developmental hourglass was not observed as in SF5. This is also consistent with the recent experimental report [36,37].

Discussions, 6th paragraph

11. **In this paper, the term ”species” and ”phylum” are used rather arbitrarily in discussing the results. It is not clear how we can define ”species” and ”phylum” in this model. In this connection, the last sentence ”This result corresponds...” seems to be sloppy.**

    The term species here means the individuals with different networks (i.e., distinct gene sets), which are branched by earlier generations in the evolution. We agree that this use is a bit sloppy, but to distinguish the comparison between different networks and between individuals of the same gene network (i.e., clones under noise), we need some term to be distinguishable from the individual. Hence, we adopt the term when necessary. Hence, we added a remark on this use in the Introduction (page 4), saying that we use the species for individuals with different GRNs branched from earlier generations.

    As for phylum, we borrowed the term, from the earlier arguments that the hourglass is seen in the species within the same phylum. We are not adopting the term straightforwardly. We remarked that the above point on the phylum is a hypothesis proposed by earlier reference, but we weakened the argument here to relax the hypothesis to make correspondence to a phylum in the Discussion (page 17-18)

Discussions, 7th paragraph.

12. **The meaning of the sentence ”when such processes...” is unclear. Since the cell interactions described in the former sentence are not investigated in this paper, more reasoning should be written for discussing them.**

    In the present study, cell-cell interaction is introduced as diffusion of chemicals (expressed proteins) as in Eq(1). Still, this is a minimal interaction as compared with the complexity in real development, so that future studies are necessary. We have revised the text accordingly.

    The last paragraph in Discussion:

    Also, only gene regulations and diffusive cellular interactions are considered. Other layers of development, such as the collective movement of cells under

mechanical forces, were reported as important in tissue morphogenesis, which is responsible for making shape, but not considered [42].

Methods: Gene expression dynamics

13. **I think r.h.s. of equation (1) may be**

$$F(i,x(r,t)) - \gamma_i x_i(r,t) + D_i \ldots$$

In eq.(1), the diffusion is expressed by the spatial second derivative. But in this model, the cell positions are discrete, and the spatial distribution of xi within cells is not considered. Thus the diffusion effect should be described by the difference between neighboring cells rather than the spatial derivative.

We have corrected the typos and revised the explanation using the discrete-space form of diffusion.

Methods: Initial/boundary conditions

14. **I could not understand the meaning of the sentence "the expressionlevels of each gene...".**

We have explicitly remarked that the initial condition is set at x_i^{init}+ variation (in the initial noise) and added the explanation, as already mentioned in the reply to 7.

Methods: Fitness function

15. **ON and OFF are defined as > 0.99 and < 0.01, respectively. Then, there should be the intermediate range of the expression. Seeing Fig 6B, expressions of some genes at the final state take intermediate values. If the final states of the target four genes are always either ON or OFF without exception, it should be mentioned. In that case, the mechanism for target genes to reach either an ON or OFF state should be described briefly. In case some exceptions have been observed, they should also be mentioned.**

The states of the four target genes almost always take ON of OFF values. We have briefly explained the reason for it in the fitness section as:

With this setup, cells are permitted to settle in intermediate states where the expression levels of the target four genes can take intermediate values, between ON and OFF. However, since gene expression sensitivity β is set to 40, which corresponds to quite high Hill coefficient, of the four target genes takes either ~1 (ON) or ~0 OFF for the final, stationary state of the developmental dynamics. In fact, only in the exceptional case in which the input to the corresponding gene is

close to the expression threshold ($\sim\frac{1}{\beta}$)), the expression level deviates from ~1 or ~0, Because the fitness postulates many cell types defined by on or off states of the target genes, such case with the intermediate expression level is not favored under the evolution with this fitness: the intermediate expression levels of the target just reduce the number of possible cell types. After evolutionary generations by the selection, such exceptional cases are hardly observed.

Methods: Mutation

16. The list of evolvable parameters is lacking.

We have added a Table to list the evolvable and fixed parameters .

Minor points:

Fig 2C and 3

17. The title of the vertical axis should be ”similarity indicator”.

We have corrected it.

Fig.5

18. The definition of the total variance in gene expressions is unclear.

It is the summation of variance over cells. We have added the explanation on it in the caption.

19. P.14 second paragraph Fig. 4 may be Fig. 5

We apologize the typos, it was Fig.3 rather than Fig 4 or 5. We have corrected it.

Fig.6

20. The definition of the variance in the timescale in C is not clear. Are these variances calculated for timescales over different cells?

Yes. We have added the explanation in the caption.

Methods: Calculation of variance...

21. *xl* should be *xi*. At the end of the paragraph, there is a summation over *j*, but I think it should be the summation over *i*.

We have corrected the typos.
